# Case Report—Pediatric Brugada Phenotype from Accident Cocaine Ingestion

**DOI:** 10.21980/J8VH28

**Published:** 2021-07-15

**Authors:** Patrick Bruss, Sarah Norris, Kaylene Pagan, Richard Cousino, Allison Grim, Gregory Reinhold

**Affiliations:** * ProMedica Monroe Regional Hospital, Department of Emergency Medicine, Monroe, MI

## Abstract

Electrocardiograms (EKGs) are rapid, non-invasive tests that provide invaluable insight into the pathologic processes plaguing our patients. We present a case of a two-year-old child found to have ingested cocaine whose EKG mimicked that of Brugada Syndrome. However, given the differences, we propose that the reason for this pattern was more likely a sodium channel toxicity secondary to the sodium channel blocking effects of cocaine, resulting in a brugada phenotype rather than a brugada genotype.

**Topics:**

Brugada, brugada phenotype, sodium channel blocker.

**Figure f1-jetem-6-3-v7:**
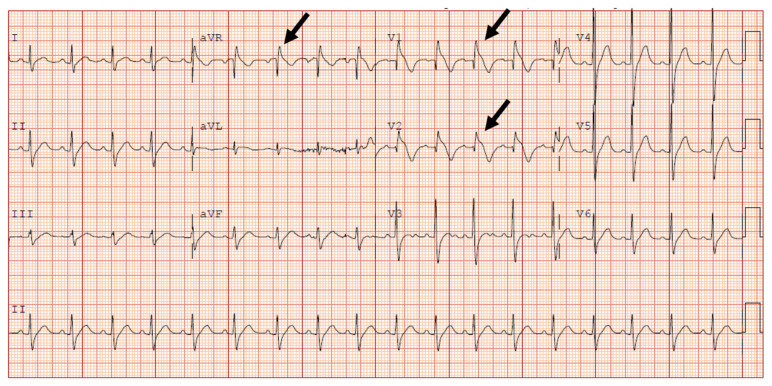
Initial electrocardiogram concerning for type I Brugada pattern. Black arrows showing incomplete right bundle branch block in V1 & ST segment elevation terminating in inverted T wave in V2. Black arrow pointing at terminal R wave present in aVR.[Fig f1-jetem-6-3-v7][Fig f2-jetem-6-3-v7][Fig f3-jetem-6-3-v7][Fig f4-jetem-6-3-v7]

**Figure f2-jetem-6-3-v7:**
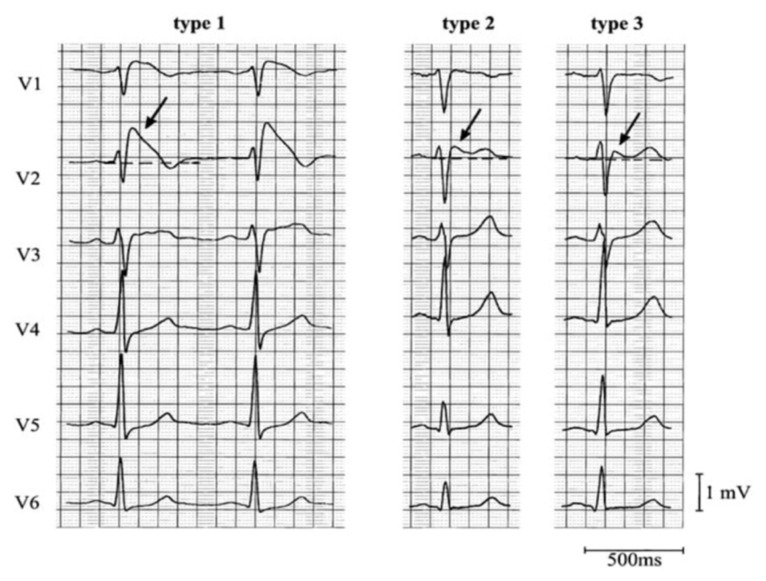
Brugada Pattern, type 1–3^9^

**Figure f3-jetem-6-3-v7:**
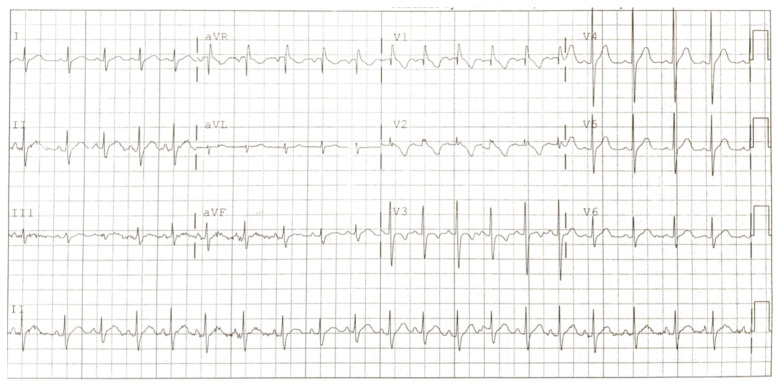
Electrocardiogram 1 month after discharge. Black arrows show persistent terminal R wave in aVR and incomplete right bundle branch block in V1. Although R wave dominance in both leads are normal in the pediatric population, incomplete bundle branch block with ST elevation resulting in inverted T wave in V1 is abnormal.

**Figure f4-jetem-6-3-v7:**
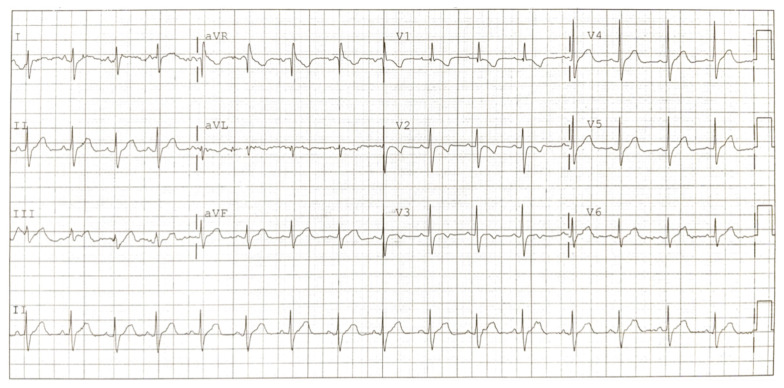
Normal electrocardiogram three months after discharge. Black arrow in V1 shows incomplete right bundle branch as well as ST elevation with terminal T wave inversion has resolved. Black arrow in V2 representative of T wave inversions in midprecordial leads (V1–3) that are normal through age 8.

## Brief introduction

Brugada Syndrome is a genetic condition that affects approximately 1/2000 individuals, predisposing them to syncope and sudden cardiac death.^1^ Individuals diagnosed with this syndrome are thought to have structurally normal hearts with a primarily electrical problem where sodium channels are blocked.^2^ Aside from a fatal, or near fatal, presentation, this syndrome can also be detected on an EKG. However, this should be done with caution since electrolyte disturbances and drugs can mimic this syndrome.^3^ Brugada Syndrome could potentially be misdiagnosed in a young individual because there are anatomical differences between an adult and child that are apparent on an EKG that can resemble this fatal syndrome.^4^ Inappropriate treatment of Brugada mimics or failure to recognize true Brugada Syndrome can both lead to significant adverse outcomes.^5^

## Presenting concerns and clinical findings

We present a case of a two-year-old male who presented to the emergency department via EMS with a complaint of lethargy. Patient was noted to be more difficult to awake that morning. EMS was called to a church where the child was with his babysitter who noted that the child had been drowsy since waking, and she had been unable to keep the patient awake during the church service. Vitals obtained by EMS were as follows: HR 112, T 98°F, 100% on room air, blood sugar 98. Of note, patient did not respond to glucose stick.

On arrival to the emergency department, the patient responded only to painful stimuli. On chart review, the patient was born via vaginal delivery at 40 weeks, weighing 3545g with APGARs (Activity, Pulse, Grimace, Appearance, and Respiration) of 8 and 5 minutes, respectively. At the time of delivery, mother’s urine drug screen (UDS) was positive only for THC. Pertinent medical history included reactive airway disease for which the patient was prescribed albuterol. No trauma or recent illness was reported. Patient has two older siblings who both have a seizure disorder. Patient attends daycare and spends half of the week with a babysitter because his mother works two jobs. Patient is up to date on vaccinations.

## Significant findings

Initial EKG was concerning for type I Brugada pattern with an incomplete right bundle branch block in V1 & ST segment elevation terminating in an inverted T wave in V2. There are also signs of sodium channel toxicity with a widened QRS complex, tachycardia and a terminal R wave present in aVR where the R wave is bigger than the S wave or the R wave is over 3mm in aVR.

## Patient course

In the department, the patient received a 200mL normal saline bolus and labs and an electrocardiogram was obtained. A point of care (POCT) glucose, POCT electrolytes, urinalysis (UA), complete blood count (CBC), complete metabolic panel (CMP), salicylate, C reactive protein (CRP) and ethanol level were obtained, all of which were unremarkable. Urine drug screen was positive for cocaine. The electrocardiogram (Figure A) demonstrated normal sinus rhythm with a right axis deviation, abnormal conduction consisting of incomplete right bundle branch block and a first-degree heart block. QRS interval was normal and ST and T waves had diffuse nonspecific changes, all concerning for Brugada Type 1.

The patient was admitted to the pediatric intensive care unit for close monitoring and escalation of care for altered sensorium secondary to ingestion of cocaine. Patient was kept NPO and started on a D5.9%NS with 20KCl infusion and was placed on continuous telemetry. EKG and neurologic exams were repeated, and social work was consulted. Poison control was contacted for further recommendations and they were skeptical of cocaine ingestion because of its short half-life. They instead suggested symptoms could be attributable to a seizure caused by a Wellbutrin ingestion, leaving the patient post-ictal. An alternative theory was that patient’s altered mental status could be a paradoxical adverse reaction of cocaine, causing sleepiness instead of hyperactivity. They recommended a repeat EKG as well as obtaining an acetaminophen and prolactin level. CT brain and lumbar puncture were not indicated after discussing the case and consulting Poison Control. Patient was observed overnight with no acute events as he was altered, but slowly returned to baseline. He was discharged home into the care of his Child Protective Services-approved guardian the following day.

The patient followed up with cardiology as an outpatient after discharge for complaint of “persistent Brugada pattern on electrocardiogram.” Patient has continued to develop well, meeting all milestones, and has had no cardiac complaints. An EKG performed just weeks after initial presentation with Brugada pattern showed persistence of pattern (Figure C). However, an EKG performed three months after incident showed normal sinus rhythm with an average heart rate of 77. There were also normal cardiac intervals and no evidence of the Brugada pattern (Figure D).

Parents and siblings also had EKGs performed for evaluation of a possible genetic disorder, which did not display any evidence of Brugada. On ECHO, patient had a structurally normal heart, trivial mitral valve insufficiency and normal biventricular systolic function. Cardiology questions whether Brugada pattern seen was indeed true Brugada or a result of the cocaine causing a sodium channel blocker toxicity. Cardiology even moved the precordial leads up one intercostal space but were still unsuccessful in attempting to elicit the pattern in the office. Plan was for a 24-hour Holter event monitor with follow-up in one year. However, the Holter event monitor testing was never completed. Since the initial presentation of possible Brugada pattern, the patient has not been evaluated for any cardiac complaints, nor was any definitive electrophysiology test was performed to confirm Brugada pattern.

## Discussion

Electrocardiograms give us great insight into underlying disease processes, but we must be mindful that there are numerous differences between the patterns seen in pediatric and adult electrocardiograms that we must consider when interpreting a patient’s electrocardiogram. The pediatric heart is initially right ventricle dominant, secondary to high pulmonary pressures, and thus produces a right axis which is normal. This axis normalizes over the first six months of life.^6^ The orientation of T waves is also important to note because T wave inversions (TWI) outside of leads aVR and V1 indicate cardiac ischemia or pulmonary emboli. However, TWI noted in the anterior precordial leads, V1–V3, are normal in a pediatric heart. These TWI are referred to as a juvenile T wave pattern and can persist into adolescence and early adulthood. There is also a dominant R wave in V1 that gradually decreases with age.^6^,^7^ This can indicate right ventricular hypertrophy in adults, but in children, this is not pathologic. Intervals of the various waves seen on an electrocardiogram can be significantly affected by medications, electrolyte abnormalities, infections, genetic conditions, and drugs. QT intervals may be difficult to categorize as normal or abnormal because this interval varies with heart rate which varies widely amongst the pediatric population.^6^ To aid practitioners, Bazett’s formula is commonly applied to determine QTc and studies have found the mean QTc to be 410msec throughout childhood.^7^ However, infants up to six months old may display what would normally be described as a prolonged QTc in adults, up to 490msec.^6^

### Sodium Channel Blockers

Sodium channel blockers include cocaine, as seen in the case presented, lidocaine, lamotrigine, procainamide, and carbamazepine. These substances inhibit sodium influx through cell membranes, thus slowing the rate and amplitude of initial depolarization, reducing cell excitability and conduction velocity.^8^ There is significant proarrhythmogenic potential when these drugs are used, including torsades de pointes. Characteristic electrocardiogram findings can be associated with their use including wide complex tachycardia, prolonged QRS and QTc, terminal R wave in aVR as well as a right axis deviation.^9^ These medications can mimic Brugada on electrocardiogram; however, they are uniquely treated more appropriately with calcium and sodium bicarbonate.

### Brugada Syndrome

Brugada Syndrome is an autosomal dominant genetic disorder caused by a defect of the gene that encodes the SCN5A sodium channel.^10^ This defect can lead to dysrhythmias including ventricular tachycardia and ventricular fibrillation, seizures, syncope, or sudden death. This syndrome generally presents itself in the fourth or fifth decade of life, affecting more men than women. There is a greater prevalence of this disorder in people of Asian descent. Brugada Syndrome can be suspected by presentation of characteristic, although potentially intermittent, electrocardiogram changes which includes ST elevation in leads V1–V3 with a complete or incomplete right bundle branch block (RBBB) appearance.^11^ Three types of Brugada have been identified. Type 1 is described as an elevated ST segment (>2mm) that descends with upward convexity to a TWI, often described as coved and is most concerning. Type 2 is described as an elevated ST segment (>1mm) that descends toward baseline then rises again to an upright T wave, often described as saddleback. Finally, Type 3 is described as an elevated ST segment (<2mm) that descends toward baseline then rises again to an upright T wave and can be coved or saddleback in appearance.^12^ Provocation testing using antiarrhythmics and ambulatory EKG monitoring can be used for diagnosis; however, definitive diagnosis is done by instilling a potent sodium channel blocker during electrophysiology studies (EPS) to precipitate Brugada or fatal dysrhythmias which are then terminated.^13^ A trial of medications such as quinidine to reduce the chance of dysrhythmias or isoprenaline for frequent life-threatening dysrhythmias has been used for treatment, although definitive treatment is with an implantable cardioverter defibrillator (ICD).^14^ Treatment is aimed at educating patients about potential triggers including avoidance of certain medications, avoiding excessive alcohol consumption and treating fevers promptly.^15^ Electrolyte abnormalities and cocaine use have been noted to mimic this disorder.

There are both benign and pathologic mimics of Brugada Syndrome that require scrutiny to determine precise etiology. Incorrect treatment and failure to recognize this syndrome could be fatal. Diagnosing this syndrome in a young child may be premature given the pediatric anatomy. However, making this diagnosis could be lifesaving if done appropriately in the absence of confounding variables. Overall, this case highlights that distinguishing Brugada from its mimics depends on several factors.

## Supplementary Information










