# Supplementary figures and images for: Case Report—Pediatric Brugada Phenotype from Accident Cocaine Ingestion
Source: J Educ Teach Emerg Med. 2021 Jul 15;6(3):V7–V12. doi: 10.21980/J8VH28 (PMC10332693; doi:10.21980/J8VH28)

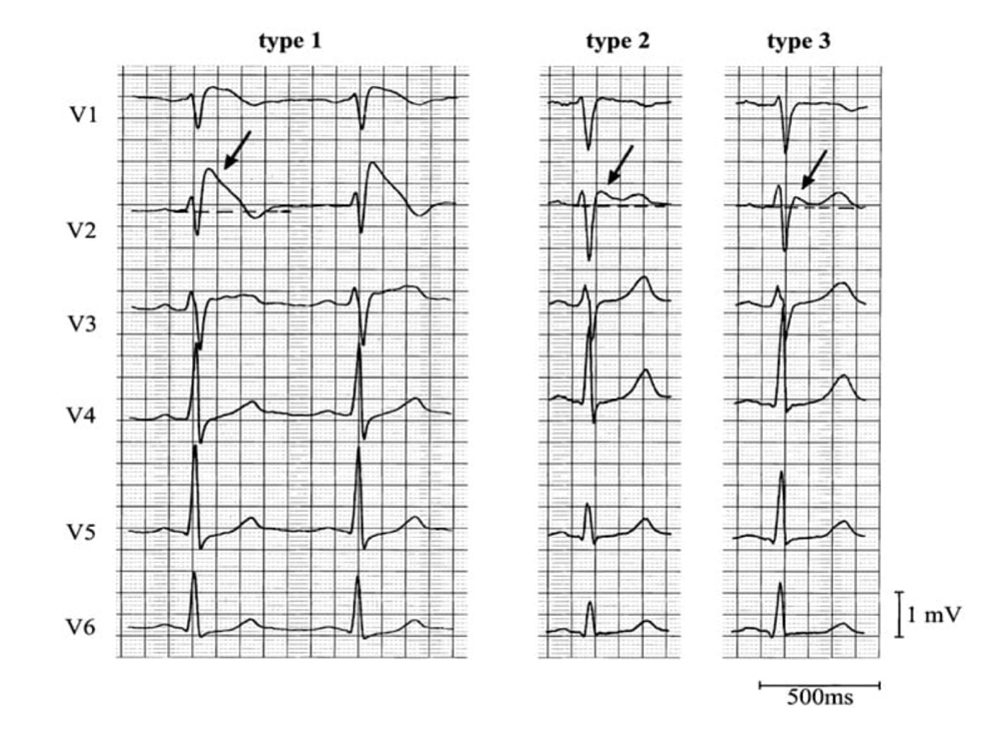

Supplement: Supplementary file 1 [file jetem-6-3-v7-supp1.jpeg]

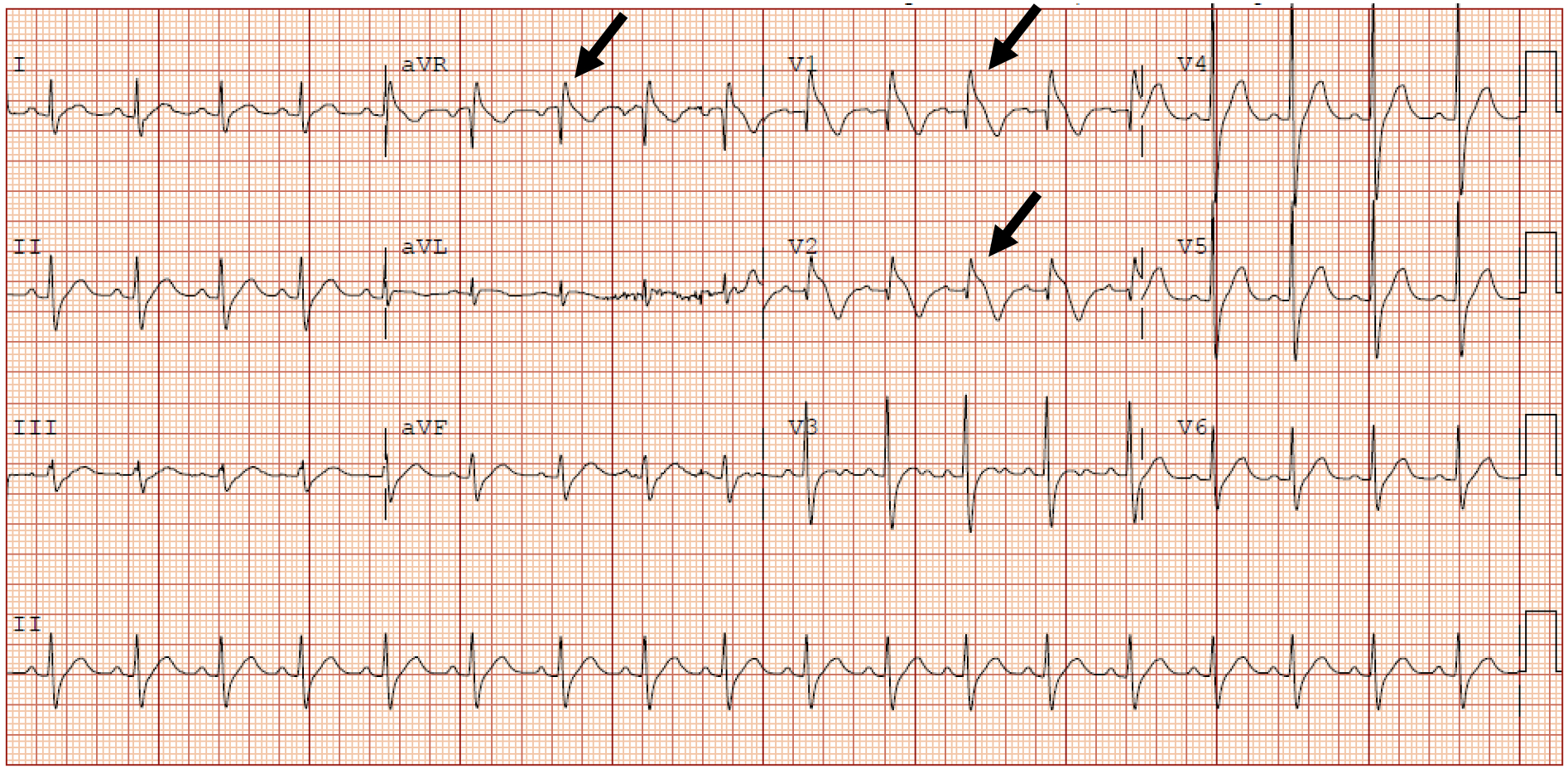

Supplement: Supplementary file 2 [file jetem-6-3-v7-supp2.jpeg]

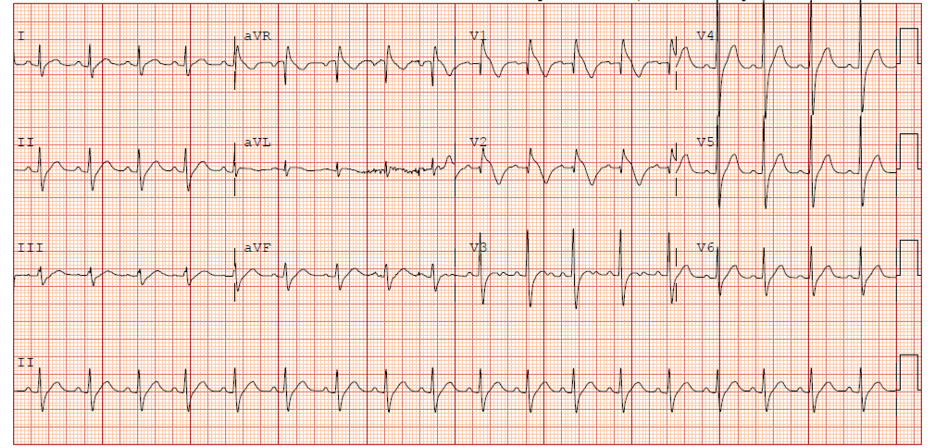

Supplement: Supplementary file 3 [file jetem-6-3-v7-supp3.jpeg]

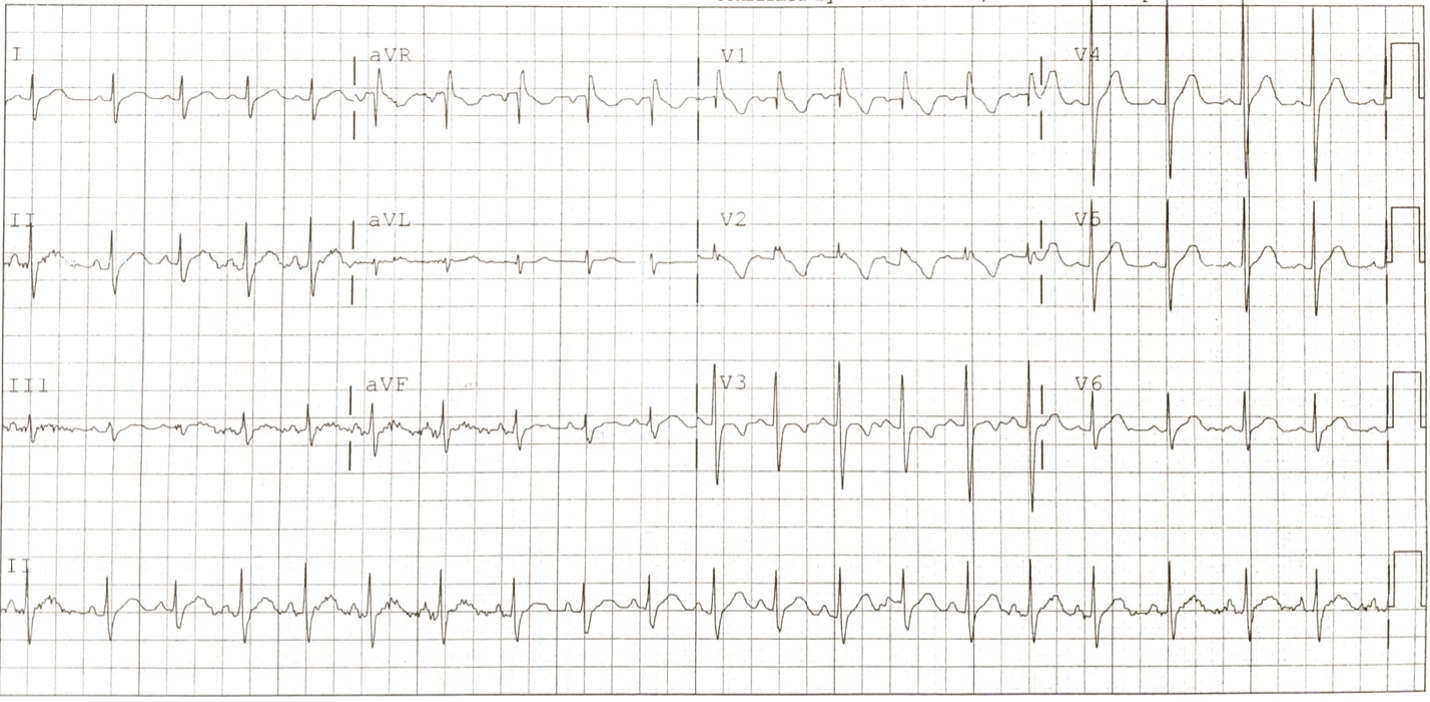

Supplement: Supplementary file 4 [file jetem-6-3-v7-supp4.jpeg]

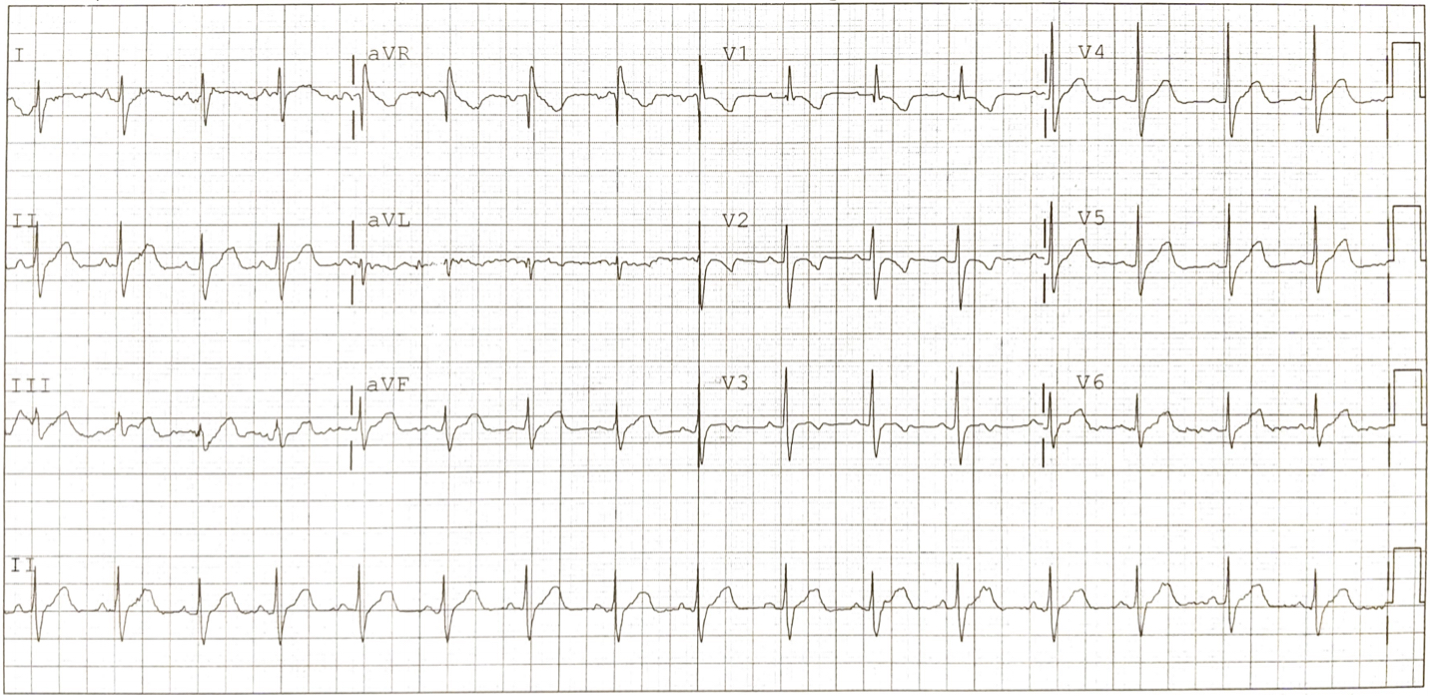

Supplement: Supplementary file 5 [file jetem-6-3-v7-supp5.jpeg]
